# Inter-facility characterization of bacteria in seafood processing plants: Exploring potential reservoirs of spoilage organisms and the resistome

**DOI:** 10.1016/j.heliyon.2024.e33866

**Published:** 2024-06-30

**Authors:** Karla Fabiola Corral-Jara, Sigurlaug Skírnisdóttir, Stephen Knobloch, Helgi Briem, José F. Cobo-Díaz, Niccolò Carlino, Pauline Bergsten, Federica Armanini, Francesco Asnicar, Federica Pinto, Avelino Alvarez-Ordóñez, Nicola Segata, Viggó þór Marteinsson

**Affiliations:** a Microbiology Research Group, Matís Ltd., C.P.113, Reykjavik, Iceland; b Department of Food Technology, Fulda University of Applied Sciences, C.P. 36037, Fulda, Germany; c Department of Food Hygiene and Technology, Universidad de León, C.P. 24071 Spain; d Segata Lab, Department CIBIO, University of Trento (UNITN), C.P. 38122, Trento, Italy; e Faculty of Food Science and Nutrition, University of Iceland, C.P. 102, Reykjavík, Iceland

**Keywords:** Fish processing plants, Gadidae, Salmonidae, Metagenome, Microbiota metabolic profile

## Abstract

A study was conducted in fish processing facilities to investigate the microbial composition, microbial metabolic potential, and distribution of antibiotic resistance genes. Whole metagenomic sequencing was used to analyze microbial communities from different processing rooms, operators and fish products. Taxonomic analyses identified the genera *Pseudomonas* and *Psychrobacter* as the most prevalent bacteria. A Principal Component Analysis revealed a distinct separation between fish product and environmental samples, as well as differences between fish product samples from companies processing either Gadidae or Salmonidae fish. Some particular bacterial genera and species were associated with specific processing rooms and operators. Metabolic analysis of metagenome assembled genomes demonstrated variations in microbiota metabolic profiles of microbiota across rooms and fish products. The study also examined the presence of antibiotic-resistance genes in fish processing environments, contributing to the understanding of microbial dynamics, metabolic potential, and implications for fish spoilage.

## Highlights

• The metagenomic analyses of seafood facilities identified control points in processing.
• Beta Diversity differences between Gadidae and Salmonidae microbiota.
• Higher ARGs on work surfaces than fish products.
• Metabolic profile differences in facility areas and among operators'equipment.
• Consistent results between MAGs and reads, enhanced study accuracy.

## Graphical abstract

**Figure:**
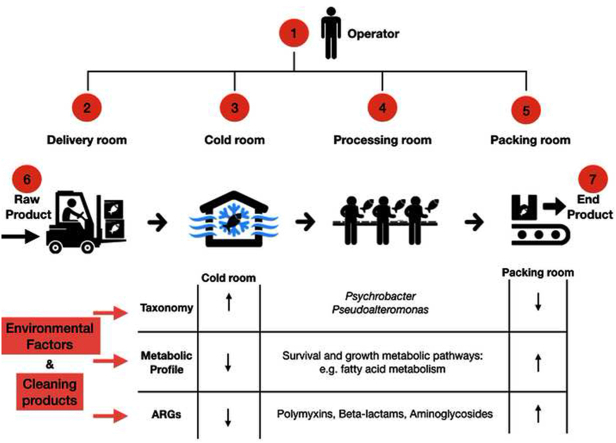

## 1 Introduction

Wild cod (*Gadus morhua*, belonging to the Gadidae family) and farmed Atlantic salmon (*Salmo salar*, belonging to the Salmonidae family) are two of the most important fish species in the seafood market, particularly in the European Union (EU) [1]. According to a report by the European Market Observatory for Fisheries and Aquaculture Products in 2019, wild cod and farmed salmon were among the top three most consumed seafood products in the EU [2].

Seafood spoilage is of significant concern due to its impact on shelf life and its implications for the global food supply. As a valuable source of protein, seafood plays a crucial role in meeting the nutritional needs of a growing global population [3]. However, microbial, enzymatic, and chemical processes can lead to spoilage, reducing the quality of seafood products [4]. This not only affects the availability of protein-rich food, but also contributes to food waste and economic losses. Additionally, while spoilage bacteria are not direct concerns for food safety, their presence may serve as indicators of potential food safety hazards if not properly managed [5].

Fish spoilage is a complex process influenced by various factors, including temperature, oxygen exposure, pH, handling and processing methods, packaging and the microbial composition of the fish product [6]. The microbiome of fish products consists of a diverse array of microorganisms, including bacteria, fungi, and viruses, which can contribute to the spoilage process. Among the microorganisms present, bacterial genera such as *Aeromonas*, *Pseudomonas*, *Photobacterium*, *Psychrobacter,* and *Shewanella* have been identified as the key players in fish spoilage [7]. Additionally, *Vibrio, Francisella, Tenacibaculum, and Flavobacterium* have been recognized as significant foodborne pathogens [8]. The relative abundance of spoilage bacterial genera may vary during storage, influenced by the storage conditions. Hygienic conditions and cleaning agents of fish processing facilities can also influence the composition of the microbiota and the extent of food spoilage [9,10].

Antibiotics are commonly used in fish farms to prevent and treat bacterial infections in farmed fish. However, this practice can lead to the development and spread of antibiotic resistance among bacteria in aquatic environments [11,12]. The resistome refers to the collection of genes that provide resistance to antimicrobial agents [13]. In the context of fish spoilage, the resistome can play a crucial role in promoting the growth of spoilage bacteria and pathogens. Fish processing facilities that are not properly cleaned and sanitized can serve as reservoirs for antimicrobial-resistant bacteria and their associated antimicrobial resistance genes (ARGs). These genes can then be transmitted to the spoilage bacteria present in the fish, increasing their resistance to antimicrobial agents and making them more difficult to control [14,15].

Understanding the dynamics and composition of the microbiota in fish products and processing facilities is crucial for developing effective strategies to prevent contamination of food products, mitigate spoilage and maintain food quality. Characterizing the microbiota in facilities handling wild cod and farmed salmon can help develop effective strategies to prevent spoilage, contributing to the growing body of knowledge on fish product microbiota and ensuring sustainable production of these valuable seafood resources by first identifying spoilage-causing bacteria to subsequently in future studies, understand spoilage mechanisms, developing preservation techniques, optimizing processing conditions and implementing quality control measures. Metagenomics is a powerful tool for studying the microbiome of fish products and processing environments. It allows for the identification of microorganisms based on their genetic material, including those that cannot be cultured [16,17]. This methodology has been used to study the composition and diversity of the microbiome in various fish products, including cod, salmon, and tuna, as well as fish processing environments [[18], [19], [20]].

The objective of this study was to conduct a comparative analysis of the taxonomic composition, ARGs distribution and microbial metabolic potential on fish products and in fish processing facilities handling either Gadidae or Salmonidae fish. By employing metagenomic analysis, the study aimed to enhance our understanding of microbiota management and control in fish processing facilities, ultimately contributing to the development of more sustainable and efficient sanitation practices. Overall, the application of metagenomics for the study of fish microbiota profiles and spoilage has the potential to make significant contributions to the seafood industry and public health.

## 2 Material and methods

### 2.1 Sample collection

In this study, a comprehensive analysis was conducted on a total of 70 samples collected from fish processing facilities. These facilities were involved in the processing of raw, gutted fish, as well as the preparation of deboned, filleted fish, and subsequent storage of the end product either frozen or fresh on ice. The samples were collected from six distinct fish production companies located in Iceland, with three companies processing Gadidae fish (cod, haddock) and three companies processing Salmonidae fish (Atlantic salmon, Arctic charr). Four negative control samples and five mock community samples were included in the analysis. Supplementary metadata providing additional details on the samples is available in Supplementary Table 1.

Environmental samples were collected from different rooms of the fish processing plant, such as the delivery room, cold room, processing and packing room. The environmental samples were classified into two groups based on their contact with fish, namely fish contact (i.e. fish containers, processing line, packaging items) and those without contact, no fish contact (i.e. floors, drains, walls). The samples were collected after the daily cleaning process in the companies. Additionally, samples were collected from the gear of operators (aprons, boots, gloves, coats), skin of raw fish (raw product) and the exterior surface of the fillet (end product). The information about the cleaning process of each company is provided in Supplementary Table 2.

Whirl-Pak polyurethane swabs (Whirl-Pak, USA) were used for collecting samples. Approximately 1 m^2^ surface area was swabbed first horizontally and then vertically, turning the swab around in between. For surfaces where swabbing 1 m^2^ was difficult or impossible, such as drains and knives, individual units, such as 1 drain or 1 knife were sampled. To decrease airborne contamination, the bag opening was kept to the side while swabbing. After taking the swab, air from the bag was removed manually before sealing it. Single-use disposable protective clothing, such as overalls, masks, gloves, footwear, and hairnets, were used during the sampling process. Ten mL of Phosphate Buffered Saline (PBS) (Sigma-Aldrich, Germany) was added to each sampling bag containing a pool of five swabs. The contents were then homogenized in a stomacher at maximum speed for 2 min, and 10 mL of homogenized liquid was recovered from each bag. This was then transferred to a sterile tube and centrifuged at 5000×*g* for 5 min at room temperature. The resulting cell pellets were stored at −80 °C until DNA extraction [21]. To avoid intentional bias in the sampling process, we have implemented random sampling techniques and established standardized protocols.

### 2.2 Metagenome sequencing

The microbial DNA from the samples was extracted using a modified version of the DNaesy PowerSoil Pro Kit (QIAGEN, Germany) according to Barcenilla et al. [21]. DNA concentrations were quantified by Qubit fluorometers (Thermo Fisher Scientific, USA) and diluted to 10 ng/μL for whole metagenome analysis. The library preparation for Illumina NovaSeq (Illumina, USA) metagenomic sequencing is based on the Illumina Nextera DNA Flex Library Prep following manufacturer's protocol. Libraries were multiplexed using dual indexing for 150 bp paired-end reads on the NovaSeq 6000 Sequencing System.

### 2.3 Metagenome analyses

#### 2.3.1 Quality control of the raw reads

The raw sequencing reads were processed using the SegataLab pipeline available at https://github.com/SegataLab/preprocessing, including chimera detection algorithms. The quality of the reads was evaluated using Trim Galore v0.6.6 (https://www.bioinformatics.babraham.ac.uk/projects/trim_galore/) and low-quality (<20), short (<75bp), and with ambiguous nucleotides (n ≥ 2) reads were discarded. Reads mapping to the human genome (hg19) and the genome sequence of the phage PhiX174 were removed using Bowtie2 [22]. To ensure the specificity of the analysis, an additional filtering step was performed using the Bowtie2 aligner and the reference genomes of relevant eukaryotic organisms including *Homo sapiens* (human), *Gadus morhua* (cod), *Melanogrammus aeglefinus* (haddock), *Salmo salar* (salmon), and *Salvelinus alpinus* (charr). The purpose of this filtering was to remove any sequences that originated from these specific eukaryotes, thus focusing the analysis specifically on microbial genomes present in the fish processing facility samples. Finally, the reads passing the quality controls were sorted and split into forward, reverse and unpaired Fastq files for each metagenome.

#### 2.3.2 Taxonomic and associated-ARGs investigation of quality-controlled reads

The taxonomic classification of reads was performed using Kraken2 software (v2.1.2) [23], with the minikraken2_v2_8 GB_201,904_UPDATE database that contains Refseq bacteria, archaea, and viral libraries. The default settings were employed, and the paired option was used to classify pre-processed R1 and R2 reads. The abundances at the species level were re-estimated using Bracken software [24], which was then merged with the Kraken2/Bracken profiles using KrakenTools.

The prediction of ARGs in the metagenomes samples was performed by employing a combination of ResFinder (https://bio.tools/resfinder) and viromeqc (http://segatalab.cibio.unitn.it/tools/viromeqc/) outputs. The sequences were first screened for the presence of known ARGs using ResFinder.

Filtered reads were aligned against the ResFinder database (downloaded on October 24, 2022) using Bowtie2 with --very-sensitive --end-to-end parameters. Obtained sam files were filtered by an in-house ruby script (https://github.com/SegataLab/MASTER-WP5-pipelines/blob/master/07-AMR_virulence_genes/count_reads.rb), which removes the gene over-estimation occurring when forward and reverse reads are aligned within the same gene. The obtained counts matrix was processed to calculate the counts per million reads (CPMs) adding a “bacterial marker” modification according to the formula:

$$CPM=\frac{\left(numberofARGreads\times Bacterialmarkersalignments\right)}{totalnumberofreads}\times 10^{6}$$

where “CPM” is the total counts per million reads value for each AMRG and “Bacterial markers alignments” is the value obtained from viromeQC, using --minlen 0 --minqual 0 parameters, in order to normalize the dataset to consider only those reads derived from bacteria.

#### 2.3.3 Reconstruction and taxonomy annotation of metagenome-assembled genomes (MAGs) with metabolic profiling on quality-controlled MAGs

To enhance the taxonomy and functional analysis performed at read level, a MAG reconstruction strategy was followed. Reads that passed quality control were assembled individually using MEGAHIT v.1.2.9 [25]. Only contigs with a minimal length of 1000 bp were kept for further analyses. The coverage distributions by mapping reads against filtered contigs was calculated using Bowtie2 with default parameters. Contigs binning was performed using MetaBAT2 [26] with the following parameters: m 1500 and --unbinned. CheckM [27] was used to assess the quality of the bins, with the following command: checkm lineage_wf -t 36 -x fa --reduced_tree. Only high-quality MAGs passing the MIMAG standard threshold (High Quality: completeness >90 % and contamination <5 %) were kept for further analysis.

The coding regions of the MAGs were extracted using Prodigal [28], following the parameters -p meta -f gff. This process aimed to identify open reading frames (ORFs) within the contigs. Subsequently, the MAGs present in each sample were subjected to grouping. To eliminate duplicate sequences, CD-HIT [29] was employed, utilizing the parameters cd-hit -c 0.9 -n 5 -d 0 -g 1 -p 1 -T 35 -M 0 -G 0 -aS 0.9 -aL 0.9. For the prediction and analysis of metabolic pathways associated with the MAGs, belonging to each sample, Anvi'o tools [30] were utilized. First, the contigs were formatted using anvi-script-reformat-fasta, and a contig database was generated using anvi-gen-contigs-database. Subsequently, the metabolic pathways were estimated using anvi-estimate-metabolism, utilizing the KEGG database (https://www.genome.jp/kegg/pathway.html) as a reference for pathway annotation.

#### 2.3.4 Statistical analysis and visualization

The taxonomic composition of the microbial community was analyzed using the phyloseq library in the R programming environment (http://www.rstudio.com/). To assess the community's structure, a Principal Component Analysis (PCoA) was performed using the ordinate function from the phyloseq R package [31]. The ordinate function was applied to the phyloseq dataset, employing the “PCoA” method and the “bray” dissimilarity metric. This analysis generated an “ordination” object, representing the ordination results based on Bray-Curtis's dissimilarity values, visually illustrating the similarities and dissimilarities between samples.

For visualization of the taxonomic profiles across the samples, a heatmap plot was generated using the hclust2 tool. The heatmap plot utilized the parameters "--f_dist_f correlation” and "--slinkage complete,” and the code can be accessed at https://github.com/SegataLab/hclust2.

To investigate set intersections and overlaps in the taxonomic profiles of the samples, the UpSetR R package was utilized [32]. The upset function from the UpSetR package facilitated the visualization and exploration of these intersections and overlaps.

Differential analysis was performed using the DESeq2 package [33]. This analysis focused on the comparison of metabolic module abundance associated with MAGs.

To ensure quality control, negative samples were included as part of the experimental and bioinformatic procedures. These negative samples were incorporated to assess and account for potential background contamination or artifacts.

## 3 Results

### 3.1 Metagenomic sequencing data retained after filtering varied by sample type

A comprehensive study was conducted, involving the collection of 70 samples (Supplementary Table 1) from six seafood processing companies in Iceland, with three companies processing Gadidae fish (cod, haddock) and three companies processing Salmonidae fish (Atlantic salmon, Artic charr). Metagenomic sequencing was performed on these samples, and the filtering of the sequencing data was carried out, with details provided in Supplementary Table 3.

The initial sequencing run generated a total of 1,311,629,636 reads (Mean 18,737,566.23; Min 110,128; Max 116,864,644), encompassing both R1 and R2 reads (2,623,259,272 combined). Following the initial quality-filtering step, 1,211,593,753 reads were deemed of sufficient quality and retained for further analysis. A subset of unpaired reads, totaling 56,218,041, was also identified.

An additional filtering step was performed to remove sequences assigned to the human and fish genomes, as well as sequences assigned to the internal control PhiX174. This step resulted in a variable number of reads retained for each individual sample, ranging from 97,350 to 222,725,274 (sum R1 + R2). The loss of total reads after the two filtering steps ranged from 1 % for surface-related samples to 99 % for fish product samples (Supplementary Table 3).

### 3.2 Differential taxonomic distribution in fish products and cold rooms among Gadidae and Salmonidae companies

A taxa heatmap was constructed to examine the relative abundance of bacterial taxa at the genus level across various sample groups, including raw products, end products, delivery rooms, cold rooms, processing rooms, packing rooms, and operators. The dendrogram revealed that the microbial community composition of packing, cold, and delivery rooms displayed a tendency to cluster together, whereas the microbial communites of operators, processing rooms, and end products formed another cluster. From the raw product, the remaining groups in the dendrogram were derived (Fig. 1A).

**Fig. 1:**
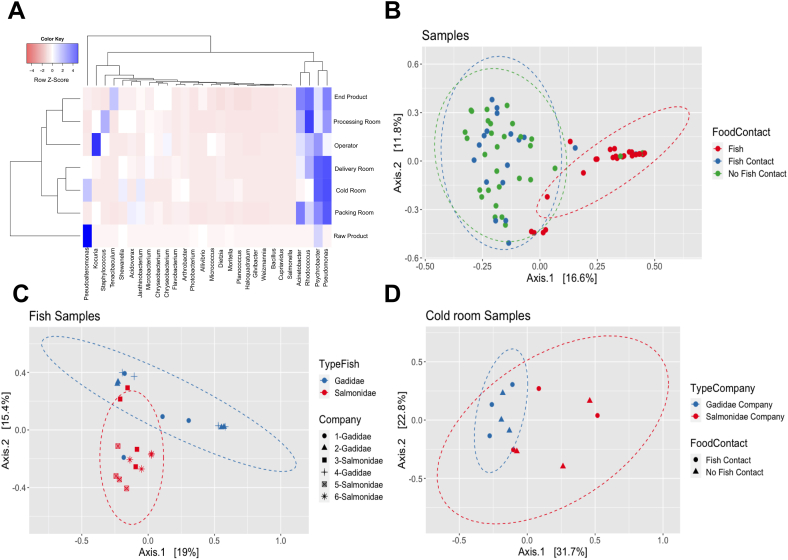
**Analysis of bacterial taxa distribution and variations in microbiota of fish processing facilities. A)** Taxa heatmap of relative abundance of bacterial taxa at the genus level across different groups. **B)** Principal Component Analysis (PCoA) between samples associated with the fish product and those from the general environment or surfaces. **C)** PCoA analysis between Gadidae and Salmonidae fish product samples. **D)** PCoA analysis between Gadidae and Salmonidae fish processing companies in the cold-rooms (*p-value* = 0.001).

To further explore the dataset and identify potential sources of variation, a PCoA was conducted. Initially, all samples were subjected to the analysis, leading to the identification of a noticeable distinction between samples related to the fish product itself and those obtained from the general environment or surfaces (fish, fish contact and no fish contact samples in Fig. 1B). Subsequent analysis focusing exclusively on the fish product samples demonstrated evident dissimilarities between the fish products of the Gadidae and Salmonidae families, as evidenced by a *permanova* analysis yielding a *p-value* of 0.001 (Fig. 1C). Furthermore, subtle variations were observed between Gadidae and Salmonidae fish processing companies in the cold-room (Fig. 1D), with a significant distinction identified by a *permanova* analysis (*p-value* = 0.001). Thus, the taxonomic composition of the microbiota profile was relatively similar across processing rooms, and dissimilarities were observed between Gadidae and Salmonidae fish companies in the cold-room and fish product. Notably, there were no significant differences in the other types of processing rooms among samples from the general environment or surfaces (Supplementary Fig. 1).

### 3.3 Association of bacterial genera and species in fish processing environments with operators

A comprehensive investigation was conducted to analyze the most abundant taxa, at the genus level, in each room and fish product samples. A schematic diagram of taxa and their abundances along the process is shown in Fig. 2A. Overall, the genus *Pseudoalteromonas* exhibited an abundance of 50 % in the raw product, while ranging from 0 % to 25 % in the other sample groups. In contrast, the genus *Psychrobacter* showed varying abundances, ranging from 6 % in the processing room to 60 % in the cold and 25 % in the delivery rooms. The genera *Kocuria* and *Micrococcus* predominated in the operator group, with an abundance of 25 %. Furthermore, the genera *Pseudomonas* (mean 17 %), *Rhodococcus* (mean 12 %), and *Acinetobacter* (mean 9 %) were found abundant across all study groups except in the raw product (Fig. 2A).

**Fig. 2:**
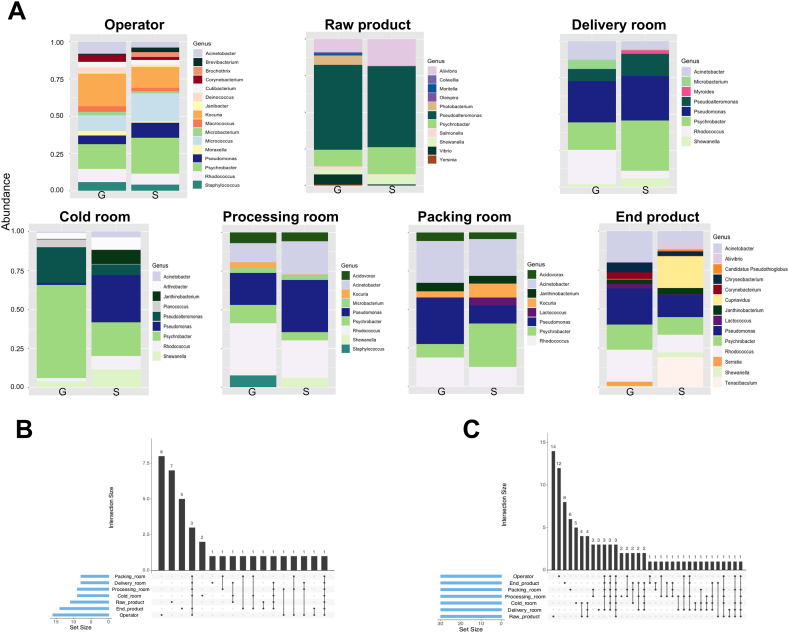
**Bacterial composition in fish processing environments. A)** Genera distribution analysis showing only the most abundant genera in fish processing rooms and fish product. G = Gadidae, S=Salmonidae. **B)** Schematic diagram illustrating the intersections between genera in each room and fish product. **C)** Schematic diagram illustrating the intersections between species in each room and fish product. Empty intersections are not shown in the figure.

*Shewanella* was detected in the samples of the end products, as well as in the delivery, cold, and processing rooms. *Allivibrio* was found in both the raw and end products. *Janthinobacterium*, also present on the end products, was identified in the cold and packing rooms. *Lactococcus* was observed on the end products and packing rooms. *Kocuria,* however, was found on the operators, processing, and packing rooms, but not on the end products. *Staphylococcus* and *Microbacterium* were detected on the operators, delivery, and processing rooms. *Corynebacterium* was identified on the end products and on the operators. Certain genera exhibited lower abundance and specificity within each room, such as *Moritella* and *Salmonella* on the raw fish, *Myroides* in the delivery room, *Planococcus* in the cold room, *Cupriavidus* on the end product and *Micrococcus* on the operators. The diversity of taxa appeared to increase in the later stages of the process, specifically starting from the processing room, and including the operators that played a role in all areas (Fig. 2A).

Comparing companies processing Gadidae and Salmonidae fish, no significant differences were observed except in the fish product itself and the cold rooms. The abundance of *Photobacterium* was higher in Gadidae fish companies, while *Aliivibrio* and *Psychrobacter* were more abundant in Salmonidae fish companies in the raw fish products. Remarkable disparities in the abundance of specific genera were found in the cold room between companies processing Gadidae and Salmonidae fish. Specifically, companies processing Gadidae fish had a higher proportion of *Pseudomonas, Psychrobacter* and *Pseudoalteromonas,* while companies processing Salmonidae fish exhibited greater abundance of *Shewanella, Rhodococcus, Arthobacter* and *Janthinobacteroum.* Regarding the end product, in Salmonidae, *Cupriavidus* and *Tenacibaculum* were found in greater proportions, while *Rhodococcus, Pseudomonas*, *Corynebacterium*, and *Chryseobacterium* were more abundant in Gadidae.

Fig. 2B provides an illustration of the intersections between genera in each room and fish product, while the same was done for intersections between species in Fig. 2C. An analysis of species distribution indicated that among the taxa found in the end product, only *Corynebacterium testudinoris* originated exclusively from the operators. *Psychrobacter* sp*. G, Psychrobacter* sp. *P11F6, Psychrobacter cryohalolentis* were found in all environments and products. Three other species, *Acinetobacter johnsonii, Rhodococcus* sp*. 008, Rhodococcus* sp*. YL-1* were identified on the operators as well as in the packing rooms, processing rooms, and delivery rooms. Three additional species, *Rhodococcus erythropolis, Pseudomonas fluorescens, Acinetobacter* sp*. TTH0-4* were present in all rooms however not on the raw fish. Out of the 30 species detected on the end products, 22 were also present in one of the rooms or on the operators. Eighteen out of the 30 species found on the operators were distributed throughout the entire process. Although nearly half of the analyzed species in the raw fish were equally distributed in the rooms, only a small number were shared with the end product, with only one species, *Aliivibrio salmonicida* shared between them.

### 3.4 Resistome composition and dynamics in fish processing facilities: Insights into ARGs distribution

In this study, it was aimed to investigate the resistome composition and diversity in fish processing facilities. By employing read level analysis, the presence and abundance of ARGs in fish product samples and samples collected from environments of different stages of fish processing were characterized.

Our findings revealed that the proportion of ARGs in end products and raw fish samples was lower in comparison to samples collected from surfaces in the fish processing environment, with the ARGs abundance in the end products the lowest (Fig. 3A). Specifically, on end product samples, ARGs exhibited a greater prevalence in Gadidae processing facilities than in those processing Salmonidae, while no statistically significant differences were present on the raw products between companies (Fig. 3B). Furthermore, through a comparative analysis of the different rooms, our study revealed that the packing room exhibited a higher ARGs abundance when compared to the cold rooms (Fig. 3C).

**Fig. 3:**
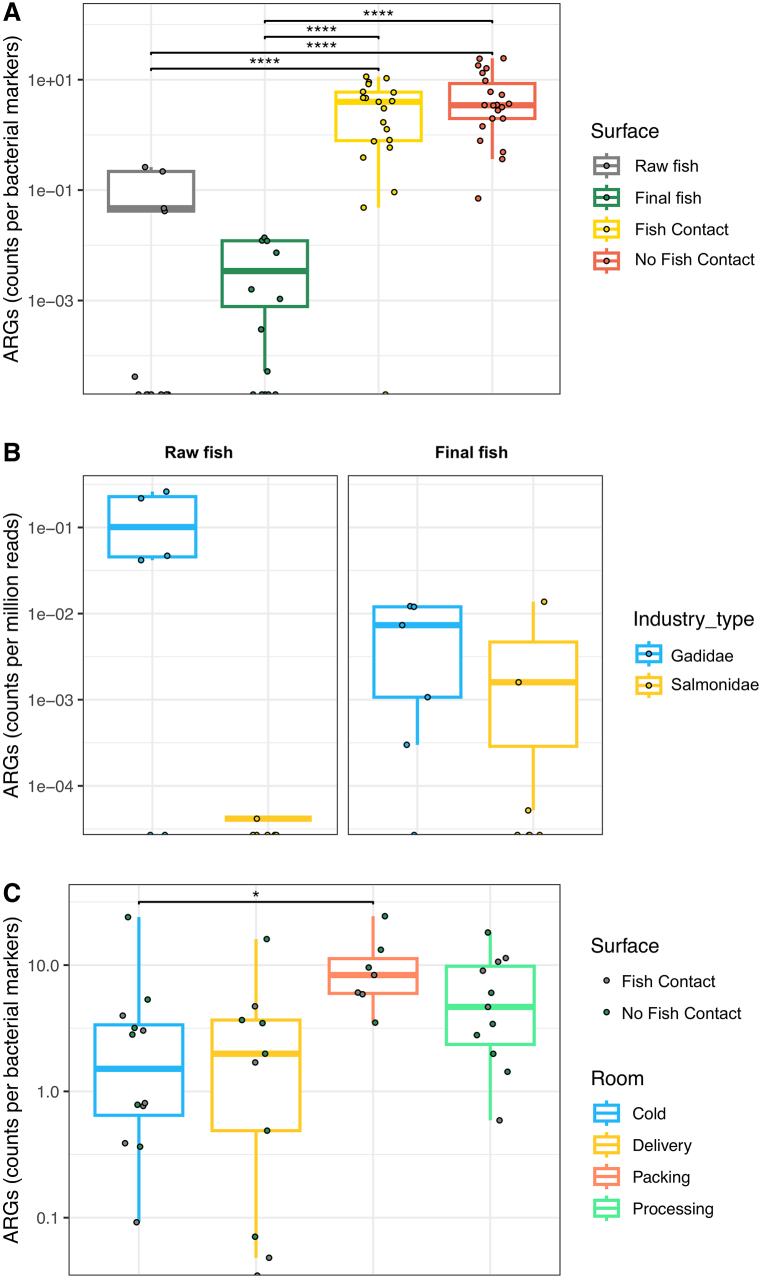
**Comparison of ARGs abundance in fish processing environments and fish product between Gadidae and Salmonidae Industries. A)** Proportion of ARGs in fish product samples and surfaces. **B)** Comparison of ARGs prevalence in raw and end product samples. **C)** Comparison of ARGs abundance between different rooms in the companies.

Upon closer examination, the investigation discerns a higher proportion of polymyxin-related ARGs on raw fish. Conversely, within the end products, predominant ARGs were associated with resistance to beta-lactams, tetracyclines, and aminoglycosides. Furthermore, in both fish contact and non-contact surfaces, ARGs related to resistance to beta-lactams and aminoglycosides were identified as the most prevalent ARGs. Furthermore, aminoglycosides and macrolides were exclusively detected on surfaces and end products, while their presence was negligible in the raw products (Supplementary Fig. 2).

### 3.5 Comparative distribution analysis of MAGs in fish processing environments

MAGs reconstruction from sequenced samples collected in various fish processing environments gave a total of 379 successfully reconstructed high-quality MAGs, representing diverse microbial taxa present in the rooms. However, no MAGs were obtained from the Salmonidae fish product samples (Supplementary Table 4). Among the MAGs retrieved, 71 bacterial genera were identified in Gadidae companies, while 69 genera were found in samples from Salmonidae companies. Table 1 presents an illustrative depiction of the distribution patterns of the main MAG genera within Gadidae and Salmonidae companies, which exhibited similarities with the analysis conducted at the read-level. Notably, *Pseudoalteromonas* MAGs were predominantly detected in both the delivery room and cold room of both Gadidae and Salmonidae companies. On the other hand, *Psychrobacter* MAGs exhibited a broader distribution across all five processing rooms in Gadidae companies. While *Psychrobacter* MAGs were more abundant in the delivery rooms of Salmonidae companies, they were also observed in the cold rooms and packing rooms. In Gadidae companies, *Kocuria* MAGs were distributed across delivery, processing and packing rooms in Gadidades, while *Kocuria* MAGs was additionally found in both the packing rooms and operator areas of Salmonidae companies. *Shewanella* MAGs were primarily detected in the cold rooms of both Gadidae and Salmonidae companies. In contrast, *Rhodococcus* MAGs were predominantly found in the processing rooms of Gadidae companies but were also present in other areas. *Rhodococcus* was distributed in the processing, packing rooms and operator areas of Salmonidae companies. The highest number of MAGs was observed in the processing room of Gadidae companies.

**Table 1:** Distribution patterns of the main MAG genera within Gadidae and Salmonidae fish processing companies.

**Genus**	**Delivery room Gadidae**	**Delivery room Salmonidae**	**Cold room Gadidae**	**Cold room Salmonidae**	**Processing room Gadidae**	**Processing room Salmonidae**	**Packing room Gadidae**	**Packing room Salmonidae**	**Operator Gadidae**	**Operator Salmonidae**
*Acinetobacter*	2	3	0	3	8	0	2	0	1	1
*Arthrobacter*	5	2	4	0	4	0	0	0	0	0
*Microbacterium*	4	0	0	0	6	2	1	0	0	2
*Rhodococcus*	3	0	1	0	10	4	2	1	1	2
*Psychrobacter*	3	2	5	1	4	0	3	1	3	0
*Kocuria*	3	0	0	0	8	0	4	2	0	2
*Pseudoalteromonas*	1	2	2	3	0	0	0	0	0	0
*Pseudomonas*	0	1	0	0	2	1	1	0	0	0
*Shewanella*	0	0	2	1	0	0	0	0	0	0
*Janthinobacterium*	0	0	0	2	2	0	1	1	0	0
*Chryseobacterium*	0	0	0	0	12	2	1	1	1	0
*Macrococcus*	0	0	0	0	2	0	0	0	2	1
*Janibacter*	0	0	0	0	2	0	0	0	0	1
*Moraxella*	0	0	0	0	0	1	0	0	1	0

The numbers denote the total count of MAGs identified in each respective environment.

Notably, 40 genera of MAGs were shared between both types of companies, as indicated by the overlapping regions in Supplementary Fig. 3A. Subsequent analysis of these 40 shared MAGs genera revealed the presence of 14 genera in the read-level analysis, including *Arthrobacter, Microbacterium, Rhodococcus, Psychrobacter, Kocuria, Acinetobacter, Pseudoalteromonas, Pseudomonas, Shewanella, Janthinobacterium, Chryseobacterium, Macrococcus, Janibacter,* and *Moraxella* (Supplementary Fig. 3B; Supplementary Table 5).

*Kaistella* species, specifically *Kaistella frigidisoli* in the cold room, *Kaistella* sp*13294015* in the processing room, and *Kaistella carnis* on the operator, were detected. *Kocuria palustris* was found in the packing room, while *Arthrobacter echini* and *Kocuria carniphila* were identified in the delivery room. Among the potential pathogenic species, *Vagococcus salmoninarum* was detected in the delivery room, while *Pseudarthrobacter ulcerisalmonis* was observed in both the cold room and packing room (Table 2).

**Table 2:**
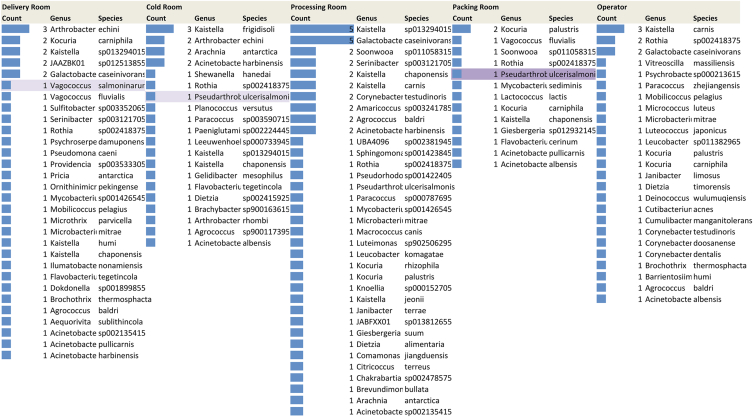
Distribution patterns of the MAG species within Gadidae and Salmonidae fish processing companies.

Pathogen or potential pathogen.

The numbers denote the total count of MAGs identified in each respective environment.

Pseudarthrobacter ulcerisalmonis as pathogen or potential pathogen.

#### 3.5.1 Comparative analysis of microbiota metabolic potential in fish processing environments

**Fig. 4:**
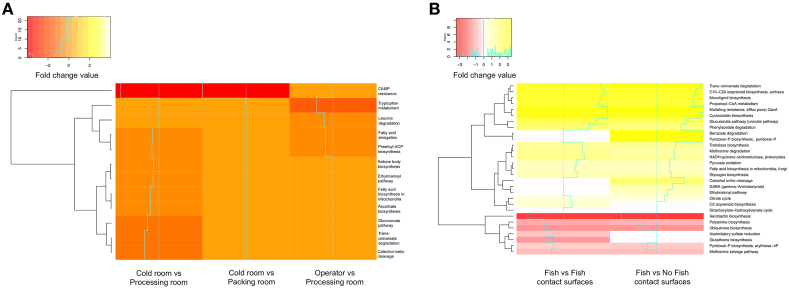
**Comparative analysis of the profiles of microbiota metabolic modules in fish product processing environments. A)** Comparisons of statistical significance: cold *vs* processing, cold *vs* packing, and operator *vs* processing **B)** Comparison between fish product samples and surfaces. The abundance of metabolic modules in the samples is visualized using a color palette, where positive fold changes are represented in a yellow palette and negative fold changes are depicted in a red-orange palette. (For interpretation of the references to color in this figure legend, the reader is referred to the Web version of this article.)

The MAGs were subjected to a metabolic profile analysis, associating the identified MAGs in a sample with different metabolic modules. Subsequently, pair-wise comparisons were conducted among the four environments of the facilities (delivery, cold, processing and packing rooms), including the operators, to investigate differences in microbiota metabolic profiles. Significant variations were observed in the metabolic profiles between the cold rooms and processing rooms, between the cold rooms and packing rooms, as well as between the operators and processing rooms (Fig. 4A). Notably, the cationic antimicrobial peptide pathway exhibited a marked negative fold change in the first two comparisons. Additionally, slight negative fold changes were observed for glucuronate metabolism, *trans*-cinnamate degradation, and catechol meta-cleavage in the comparison between the cold room and processing room. Moreover, a slight negative fold change was observed in tryptophan metabolism on the operator compared to the processing rooms. The abundance of metabolic modules associated with fatty acid metabolism, ascorbate metabolism, and ketone body biosynthesis was lower in the cold rooms, with a fold change close to zero (Fig. 4A).

In the comparative metabolic profile analysis between fish product samples and surfaces either in contact or not in contact with the fish, distinct microbial metabolic potentials were observed in the bacterial communities. Bacteria associated with the fish products exhibited positive fold changes in various pathways. These included fatty acid biosynthesis, citrate cycle, propanoyl CoA metabolism, glucuronate pathway, pyruvate oxidation, and glycogen biosynthesis. Conversely, certain metabolic pathways displayed a negative fold change in the fish-associated bacteria. These pathways included aerobactin synthesis, polyamine metabolism, ubiquinone synthesis, glutathione metabolism, pyridoxal phosphate biosynthesis, and methionine salvage (Fig. 4B).

## 4 Discussion

The primary objective in well-managed fish processing facilities is to minimize the presence of bacteria to negligible levels on the end product [34]. However, complete eradication of bacteria from the fish processing environment is challenging due to their ubiquitous nature. Despite stringent control measures, a certain level of bacterial presence may persist and has shown to be present in Icelandic fisheries processing plants [35]. Therefore, the focus shifts towards minimizing the presence and growth of harmful bacteria, particularly spoilage and foodborne pathogens, through effective control measures.

Fish processing companies must employ proper handling, processing, and storage practices, maintain temperature controls, conduct regular monitoring and testing, and adhere to good manufacturing practices (GMP) and Hazard Analysis and Critical Control Points principles to ensure the production of safe and high-quality fish products [[36], [37], [38]]. Strict hygiene practices, robust cleaning and sanitization protocols significantly reduce the risk of bacterial contamination. As an example, in a recent study by Moreira et al., researchers explored surface modification of stainless steel as a means of enhancing cleanliness. Their findings indicated that incorporating silicon (a-C:H:Si or SICAN) into the stainless steel surface significantly increased the efficacy of a chlorine-based cleaning protocol in reducing *Escherichia coli* counts compared to untreated stainless steel [39].

This investigation yielded significant findings regarding the composition of microbial communities in seafood processing environments. Variations in bacterial presence and distribution throughout the different stages of the processing chain in both Gadidae and Salmonidae processing facilities were observed through the analysis of metagenomic data. Notably, the analysis at both the read levels and MAGs produced similar results, particularly in identifying the key bacterial genera and their distribution within the rooms. Integrating information from both approaches enabled a more detailed characterization not only of the microbial community's structure but also of the microbial metabolic potential, and the distribution of ARGs in each processing room of the fish processing facilities, enhancing the resolution and accuracy of the results.

While MAGs provide insights into the genetic content of individual microbial genus and species, they may not capture the entire microbial community due to assembly limitations [40]. On the other hand, read-level analysis allows for the identification of a broader range of taxa and can be more sensitive and specific in certain cases [41]. Among the bacterial genera identified in both types of analysis, notable examples included *Psychrobacter, Pseudomonas, Shewanella*, and *Moraxella*, which are frequently linked to the spoilage of fish and seafood products. These bacteria thrive at refrigeration temperatures and are notorious for causing off-flavors, off-odors, and slime formation, all of which contribute to the decline in fish quality, resulting in reduced shelf life and decreased consumer acceptability [42].

The differences between Gadidae and Salmonidae fish processes and the intrinsic microbiota can be associated with each type of fish when establishing control protocols [43,44]. These variations influence the development and spread of microbiological hazards in fish processing facilities. By understanding and addressing these differences, tailored control measures can be implemented to mitigate the risk of contamination and preserve the quality of the end products.

Our study observed variations in the relative abundances of bacteria, such as *Pseudomonas, Acinetobacter, Shewanella,* and *Psychrobacter* in the end product, where they were found to be more abundant in Gadidae fish, except for *Shewanella,* which was more prevalent in Salmonidae. Previous studies have consistently found *Shewanella* species to be frequently isolated from salmon, indicating their affinity for this fish species. For example, Moretro et al. identified *Shewanella putrefacien*s as a predominant spoilage bacterium in salmon fillets and processing plants [45].

One contributing factor to the observed differences in bacterial proportions is the distinction between wild-caught (Gadidae) and farmed fish (Salmonidae). Wild-caught fish are subject to natural environmental conditions and variations in the microbial communities present in their habitats. In contrast, farmed fish are typically reared in controlled aquaculture systems with regulated environmental conditions, potentially exposing them to different bacterial species [46,47]. These differences in microbial exposure can lead to distinct bacterial profiles observed in wild-caught versus farmed fish.

Furthermore, the temperature in fish processing cold rooms directly affects product quality and shelf life. Variations in storage conditions between Gadidae and Salmonidae companies can influence the proportions of spoilage bacteria, such as the higher proportion of *Psychrobacter* in Gadidae companies, observed in our data. Cold-water fish like cod are typically processed at temperatures between −1 and 2 °C, while salmon is processed at temperatures ranging from 0 to 4 °C. These temperature ranges are based on scientific evidence, industry practices, and regulatory guidelines for optimal fish preservation and quality [48,49].

This study suggested that bacterial populations on the fish can primarily result from the operator's practices and the operations they execute, although high hygiene standards were employed in all of the companies. The microbial composition of raw fish differed from the final fish product, in both companies, suggesting that the contamination source in the end product may come from the work environments. This finding aligns with previous research emphasizing the significant contribution of human operators to microbial contamination in food processing environments. Transfer of microorganisms by personnel particularly from hands, is of vital importance [50,51]. During handling and preparation, bacteria are transferred from contaminated hands of food workers to food and to other surfaces [52]. Poor hygiene, particularly deficient or absence of hand washing has been identified as the causative mode of transmission [53]. Proper hand washing and disinfection has been recognized as one of the most effective measures to control the spread of pathogens, especially when considered along with the restriction of ill workers [51].

In the fish processing plants that we sampled, all operators followed strict hygiene protocols, including wearing disposable or multi-use gloves, clean or disposable aprons, and appropriate footwear and clothing such as shoes/boots, overalls, or shirt/coats [21]. While both types of companies sampled in this study utilized sodium hydroxide and sodium hypochlorite in their cleaning routines, cod companies employed additional substances like sulfonic acids, amines, isopropylalcohol, ammonia, sodium metasilicate pentahydrate, sodium alkylbenzene sulfonate, thoxylated C9-11 alcohol. In contrast, the salmon processing companies incorporated ortofosforic acid, and dodesulbensens-sulfuric acid.

These differences in the use of cleaning products could also lead to differences in taxonomic profiles. For example, hydrogen peroxide and sodium hypochlorite disinfectants are more effective against *Staphylococcus aureus* and *Pseudomonas aeruginosa* biofilms than quaternary ammonium compounds *in vitro* [54].

Moreover, our study predicted insights into the metabolic potential in bacteria within the different rooms of fish processing facilities. Notable differences in the abundance of metabolic modules suggested potential adaptations of bacteria to the environment. Our findings suggested that there exist areas characterized by higher metabolic potential in bacteria, such as the packing room and those involving the operators, as well as the end product itself. They exhibited increased presence of metabolic pathways including fatty acid biosynthesis, citrate cycle, propanoyl CoA metabolism, glucuronate pathway, pyruvate oxidation, and glycogen biosynthesis. These pathways play crucial roles in energy production, nutrient storage, and the synthesis of vital cellular components in the microbiota and could be related with the metabolic mechanisms of spoilage bacteria. *Shewanella putrefaciens* (*00A and 00B*) isolated from chilled spoiled bigeye tuna showed that the upregulated intracellular proteins were mainly involved in sulfur metabolism, amino acid metabolism, and aminoacyl-tRNA biosynthesis, while downregulated proteins were related to propanoate metabolism. In contrast, extracellular proteins were mainly involved in ribosomes, quorum sensing, and carbohydrate metabolism [55].

Contrary, glucuronate metabolism, *trans*-cinnamate degradation, and catechol meta-cleavage, fatty acid metabolism, ascorbate metabolism, and ketone body biosynthesis were found diminished in the cold room, suggesting lower microbial metabolism at low temperatures, Although high taxonomic diversity can exist, the functional diversity in bacteria may be reduced under low temperatures and specific environmental conditions [56]. It has been reported for instance, that *Psychrobacter arcticus 273-4* species exhibit lower metabolic rates at low temperatures compared to mesophilic bacteria, attributed to the downregulation of specific metabolic pathways, such as genes for transcription, translation, energy production and most biosynthetic pathways. This downregulation allows *Psychrobacter* to conserve energy and prioritize essential cellular functions for survival in cold environments [57]. Moreover, the cationic antimicrobial peptide pathway, involved in antimicrobial defense, showed a marked negative fold change in the cold room compared to the processing and packing room, indicating an impact on antimicrobial defense gene expression.

Spoilage bacteria, although primarily associated with food quality deterioration rather than causing diseases, can serve as potential reservoirs and vectors for ARGs, which would translate into a problem for the food industry [58]. This fact indicates the importance of studying the profile of ARGs in the different fisheries processes.

A difference in the proportion of ARGs, was observed between the cold room and the packing room, with a greater abundance of ARGs in the packing room. Overall, while cold temperatures themselves may not directly influence ARGs expression, they play a significant role in shaping the microbial ecology and environmental conditions within fishery cold rooms, ultimately affecting antibiotic resistance in the microbiota. Temperature is a key factor that affects the survival of bacteria in the presence of antibiotics, temperature can alter cellular behavior and lead to antibiotic tolerance and persistence [59].

In the final fish product, fewer ARGs were detected compared to both the surfaces of the built environment with or without contact to the fish. Similarly, the raw fish product exhibits fewer ARGs compared to these surfaces. This observation may be attributed to the increased exposure of surfaces to detergents or cleaning products during cleaning procedures. Although often overlooked, the exacerbated use of disinfectants can lead to antimicrobial resistance and this may exacerbate resistance to antibiotics [60]. It is recognized that certain products utilized in the disinfection processes within the fisheries examined in our study contribute to the proliferation of ARGs. For instance, previous reports have indicated a positive correlation between the antimicrobial triclosan and *erm(X)* gene, a 23S rRNA methyltransferase known for conferring resistance to multiple antibiotics [61]. Similarly, studies have shown that the disinfection with byproducts, such as chlorite and iodoacetic acid, exhibit antibiotic-like effects, leading to the evolution of resistant *Escherichia coli* strains under high and low exposure concentrations [62].

The profile of ARGs observed in raw fish significantly differed from that of the surfaces of the environment, in contact or not with fish, and the end products in both companies. In contrast, the ARGs profiles of the surfaces and end products exhibited greater similarity to each other, effect similarly observed with taxonomic distribution in our study. Among the ARGs identified, those belonging to the families of polymyxins, beta-lactams, aminoglycosides, and tetracyclines were found to be the most abundant. These findings emphasized the potential role of processing environments as a source of antimicrobial-resistant microorganisms that can impact the final product in fish processing companies.

To delve deeper into the molecular mechanisms underlying the factors mentioned above, our attention will be directed towards the cold room, a critical juncture in the fish processing chain. Additionally, it's worth noting that bacteria's adaptive response to different food-related stresses, particularly cold temperatures, has been demonstrated to provide cross-protection against antibiotics. This phenomenon could potentially expedite the spread of antibiotic resistance throughout the food chain [58].

The cold environment reduces bacterial metabolic rates, leading to a narrower range of functional activities and potentially limiting their contributions. The lower metabolic activity of bacteria in cold rooms can impact their response to antibiotics. Dormant or less active bacterial cells are generally less susceptible to the effects of antibiotics, reducing selective pressure on the bacterial population and potentially influencing the evolution and maintenance of antibiotic resistance genes within the microbiota. Tolerant bacteria often exist in a dormant state, neither growing nor dying but resisting antibiotic stress until they can reawaken once the stress is gone. Tolerance has been linked to the spread of antibiotic resistance [63]. It has been reported that an increased metabolism restores the susceptibility of tolerant bacteria to antibiotics [64]. Understanding the interplay between temperature, cleanliness, and spoilage bacterial metabolism is crucial for elucidating the dynamics of antimicrobial resistance in the fish microbiota and implementing effective mitigation strategies to prevent the spread of ARGs in food processing settings.

Our study had some limitations that should be acknowledged. Firstly, increasing the number of samples collected from each sampling site and including a broader representation of fish producers would have been preferable. Furthermore, we were unable to conduct comparisons between our analyzed samples and those sourced from fisheries in regions beyond Iceland. To address these limitations, future research could involve longitudinal studies to monitor alterations in resistome composition and microbial community dynamics over time across global fish processing facilities. It should also be noted that the overall bacterial load and number of reads from the final product was much lower than those found in the other samples, resulting in a smaller data set and potentially a larger source for errors due to random events that effect the presence of certain taxa and metabolic pathways.

## 5 Conclusion

The presence of live spoilage and pathogenic bacteria throughout the processing facility can have significant implications for seafood production. These microbes can potentially contaminate the end product, leading to quality deterioration and potential health risks for consumers. Incorporating metagenomic sequencing into a hygiene plan can offer several benefits for seafood producers, safety regulators, and customs officials. This study represents an important contribution to the field, as it applies environmental sequencing techniques to seafood producing facilities. One potential future task is the establishment of new or additional critical control points for processing fish based on the identified microbial risks. These control points can target specific areas prone to contamination or focus on critical steps in the production process to minimize the introduction and spread of spoilage organisms and pathogens. Regular or annual testing can also be implemented to monitor changes in the microbial composition and evaluate the effectiveness of control measures, such as cleaning and disinfection, over time, allowing for adjustments and improvements as needed. Continued research and application of these techniques will contribute to the development of more robust and effective control strategies, ultimately ensuring the production of safe and high-quality seafood products. One prospective strategy involves constructing an *in silico* model that integrates taxa, ARGs, and metabolic pathways, incorporating external factors such as temperature variations, cleaning products, and operator practices to generate predictive insights.

## Funding

The European Commission's 10.13039/501100007601Horizon 2020 Research and Innovation Programme financed the MASTER project (Microbiome Application for Sustainable Food Systems via Technologies and 10.13039/100005721Enterprise) (Grant Agreement No. 818368).

## CRediT authorship contribution statement

**Karla Fabiola Corral-Jara:** Writing – original draft, Visualization, Formal analysis, Conceptualization. **Sigurlaug Skírnisdóttir:** Writing – review & editing, Methodology, Funding acquisition, Conceptualization. **Stephen Knobloch:** Writing – review & editing, Methodology, Funding acquisition, Conceptualization. **Helgi Briem:** Writing – review & editing, Software, Formal analysis, Data curation, Conceptualization. **José F. Cobo-Díaz:** Writing – review & editing, Formal analysis. **Niccolò Carlino:** Writing – review & editing, Methodology. **Pauline Bergsten:** Formal analysis. **Federica Armanini:** Methodology. **Francesco Asnicar:** Data curation. **Federica Pinto:** Methodology. **Avelino Alvarez-Ordóñez:** Writing – review & editing, Funding acquisition. **Nicola Segata:** Writing – review & editing, Supervision, Funding acquisition. **Viggó þór Marteinsson:** Writing – review & editing, Supervision, Methodology, Funding acquisition, Conceptualization.

## Declaration of competing interest

The authors declare that they have no known competing financial interests or personal relationships that could have appeared to influence the work reported in this paper.

## Figures and Tables

**figs1:**
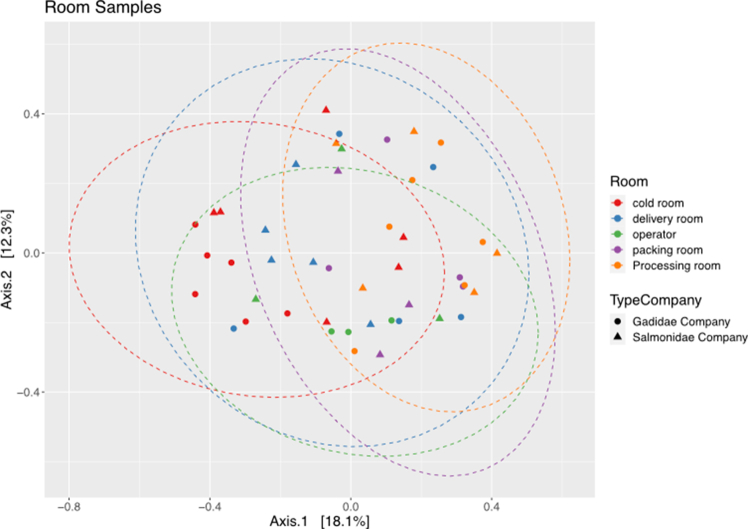


**figs2:**
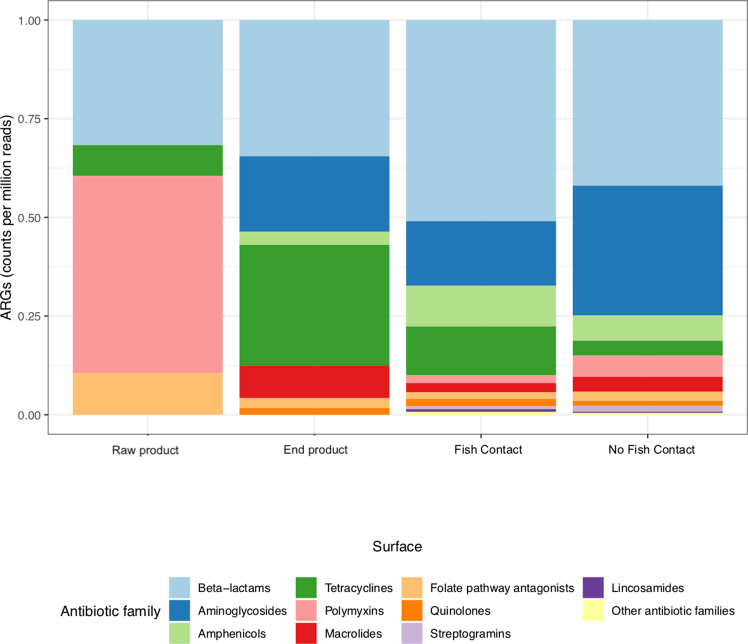


**figs3:**
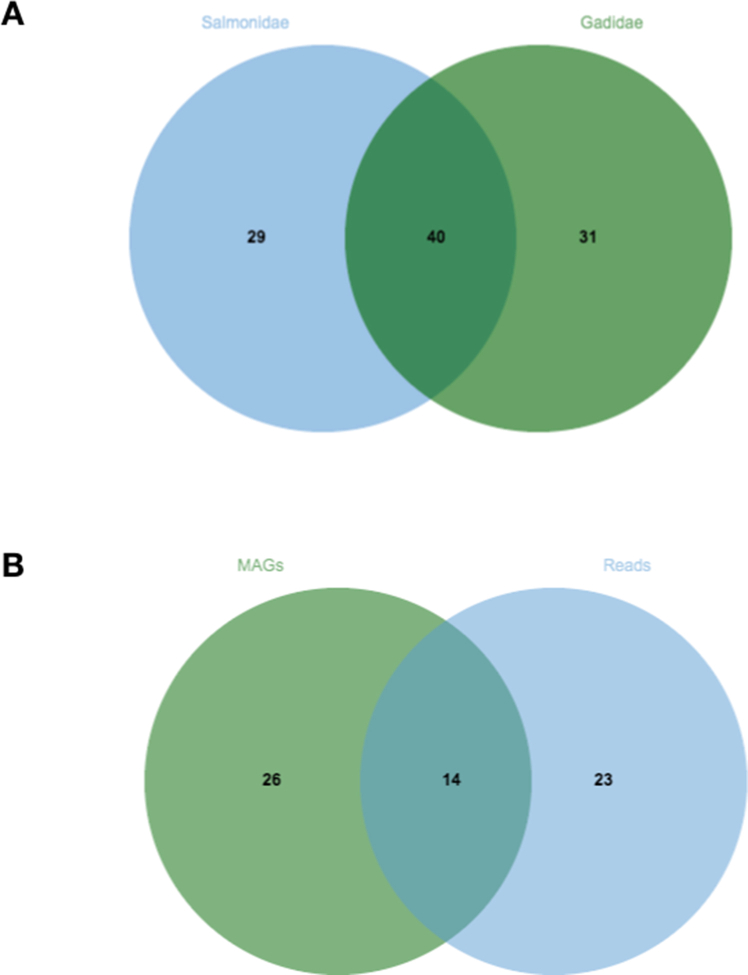


## References

[1] FAO (2020, 2020).

[2] (2020). European market observatory for fisheries and aquaculture products. The EU fish market.

[3] FAO. Food and Agriculture Organization of the United Nations (2018).

[4] Gram L., Huss H.H. (1996).

[5] Odeyemi O.A., Alegbeleye O.O., Strateva M., Stratev D. (2020). Understanding spoilage microbial community and spoilage mechanisms in foods of animal origin. Compr. Rev. Food Sci. Food Saf..

[6] Lougovois V.P., Kyrana V.R., Riley A.P. (2005). Food Policy, Control, and Research.

[7] Zhuang S., Hong H., Zhang L., Luo Y. (2021). Spoilage-related microbiota in fish and crustaceans during storage: research progress and future trends. Compr. Rev. Food Sci. Food Saf..

[8] Drønen K., Roalkvam I., Nilsen H., Olsen A.B., Dahle H., Wergeland H. (2022). Presence and habitats of bacterial fish pathogen relatives in a marine salmon post-smolt RAS. Aquac Rep.

[9] Sheng L., Wang L. (2021). The microbial safety of fish and fish products: recent advances in understanding its significance, contamination sources, and control strategies. Compr. Rev. Food Sci. Food Saf..

[10] Elsheshtawy A., Clokie B.G.J., Albalat A., Nylund A., Isaksen T.E., Napsøy Indrebø E., Andersen L., Moore L.J., MacKenzie S. (2023). Net cleaning impacts Atlantic salmon gill health through microbiome dysbiosis. Frontiers in Aquaculture.

[11] Pepi M., Focardi S. (2021). Antibiotic-resistant bacteria in aquaculture and climate change: a challenge for health in the mediterranean area. Int. J. Environ. Res. Publ. Health.

[12] Okeke E.S., Chukwudozie K.I., Nyaruaba R., Ita R.E., Oladipo A., Ejeromedoghene O., Atakpa E.O., Agu C.V., Okoye C.O. (2022). Antibiotic resistance in aquaculture and aquatic organisms: a review of current nanotechnology applications for sustainable management. Environ. Sci. Pollut. Control Ser..

[13] Singh S., Verma N., Taneja N. (2019). The human gut resistome: current concepts & future prospects. Indian J. Med. Res..

[14] Marijani E. (2022). Prevalence and antimicrobial resistance of bacteria isolated from marine and Freshwater fish in Tanzania. Internet J. Microbiol..

[15] Ballash G.A., Baesu A., Lee S., Mills M.C., Mollenkopf D.F., Sullivan S.P.M., Lee J., Bayen S., Wittum T.E. (2022). Fish as sentinels of antimicrobial resistant bacteria, epidemic carbapenemase genes, and antibiotics in surface water. PLoS One.

[16] Liu S., Moon C.D., Zheng N., Huws S., Zhao S., Wang J. (2022). Opportunities and challenges of using metagenomic data to bring uncultured microbes into cultivation. Microbiome.

[17] Billington C., Kingsbury J.M., Rivas L. (2022). Metagenomics approaches for improving food safety: a review. J. Food Protect..

[18] Trujillo-González A., Li T., Potts J., Nicol S., Allain V., Godwin S.C., Vourey E., Portal A., Kumasi B., Usu T., Rodrigo A., Gleeson D. (2022). Can Stomach content and microbiomes of tuna provide near Real-time detection of Ecosystem composition in the Pacific ocean?. Front. Mar. Sci..

[19] Rasmussen J.A., Villumsen K.R., Duchêne D.A., Puetz L.C., Delmont T.O., Sveier H., Jørgensen L. von G., Præbel K., Martin M.D., Bojesen A.M., Gilbert M.T.P., Kristiansen K., Limborg M.T. (2021). Genome-resolved metagenomics suggests a mutualistic relationship between *Mycoplasma* and salmonid hosts. Commun. Biol..

[20] Riiser S., Haverkamp T.H.A., Varadharajan S., Borgan Ø., Jakobsen K.S., Jentoft S., Star B. (2020).

[21] Barcenilla C., Cobo-Díaz J.F., De Filippis F., Valentino V., Cabrera Rubio R., O'Neil D., Mahler de Sanchez L., Armanini F., Carlino N., Blanco-Míguez A., Pinto F., Calvete-Torre I., Sabater C., Delgado S., Ruas-Madiedo P., Quijada N.M., Dzieciol M., Skírnisdóttir S., Knobloch S., Puente A., López M., Prieto M., Marteinsson V.T., Wagner M., Margolles A., Segata N., D. Cotter P., Ercolin D., Alvarez-Ordóñez A. (2024). Improved sampling and DNA extraction procedures for microbiome analysis in food-processing environments. Nat. Protoc..

[22] Langmead B., Salzberg S.L. (2012). Fast gapped-read alignment with Bowtie 2. Nat. Methods.

[23] Wood D.E., Lu J., Langmead B. (2019). Improved metagenomic analysis with Kraken 2. Genome Biol..

[24] Lu J., Breitwieser F.P., Thielen P., Salzberg S.L. (2017). Bracken: Estimating species abundance in metagenomics data. PeerJ Comput Sci.

[25] Li D., Luo R., Liu C.M., Leung C.M., Ting H.F., Sadakane K., Yamashita H., Lam T.W. (2016). MEGAHIT v1.0: a fast and scalable metagenome assembler driven by advanced methodologies and community practices. Methods.

[26] Kang D.D., Li F., Kirton E., Thomas A., Egan R., An H., Wang Z. (2019). MetaBAT 2: an adaptive binning algorithm for robust and efficient genome reconstruction from metagenome assemblies. PeerJ.

[27] Parks D.H., Imelfort M., Skennerton C.T., Hugenholtz P., Tyson G.W. (2015). CheckM: assessing the quality of microbial genomes recovered from isolates, single cells, and metagenomes. Genome Res..

[28] Hyatt D., Chen G.-L., Locascio P.F., Land M.L., Larimer F.W., Hauser L.J. (2010). Prodigal: prokaryotic gene recognition and translation initiation site identification. http://www.biomedcentral.com/1471-2105/11/119.

[29] Fu L., Niu B., Zhu Z., Wu S., Li W. (2012). CD-HIT: accelerated for clustering the next-generation sequencing data. Bioinformatics.

[30] Eren A.M., Esen O.C., Quince C., Vineis J.H., Morrison H.G., Sogin M.L., Delmont T.O. (2015). Anvi'o: an advanced analysis and visualization platform for omics data. PeerJ.

[31] McMurdie P.J., Holmes S. (2013). Phyloseq: An R package for Reproducible Interactive Analysis and Graphics of Microbiome Census Data. PLoS ONE.

[32] Conway J.R., Lex A., Gehlenborg N. (2017). UpSetR: an R package for the visualization of intersecting sets and their properties. Bioinformatics.

[33] Love M.I., Huber W., Anders S. (2014). Moderated estimation of fold change and dispersion for RNA-seq data with DESeq2. Genome Biol..

[34] Kontominas M.G., Badeka A.V., Kosma I.S., Nathanailides C.I. (2021). Innovative seafood preservation technologies: recent developments. Animals.

[35] Reynisson E., Magnússon S.H., Rúnarsson Á.R., Marteinsson V. Bacterial diversity in the processing environment of fish products, Matís report 11-20 (Mars 2010). https://matis.is/wp-content/uploads/skyrslur/11-10-Lokaskyrsla-1790.pdf.

[36] Bedane T.D., Agga G.E., Gutema F.D. (2022). Hygienic assessment of fish handling practices along production and supply chain and its public health implications in Central Oromia, Ethiopia. Sci. Rep..

[37] Seafood HACCP Alliance for Training (2000).

[38] Oliveira R.S., Rodrigues M.J., Henriques A.R. (2021). Specific hygiene procedures and practices assessment: a cross-sectional study in fresh fishery product retailers of lisbon's traditional food markets. Foods.

[39] Moreira J.M.R., Fulgêncio R., Alves P., Machado I., Bialuch I., Melo L.F., Simões M., Mergulhão F.J. (2016). Evaluation of SICAN performance for biofouling mitigation in the food industry. Food Control.

[40] Meziti A., Rodriguez-R L.M., Hatt J.K., Peña-Gonzalez A., Levy K., Konstantinidis K.T. (2021). The reliability of metagenome-assembled genomes (MAGs) in representing natural populations: insights from comparing MAGs against isolate genomes derived from the same fecal sample microbial ecology.

[41] Peker N., Garcia-Croes S., Dijkhuizen B., Wiersma H.H., Van Zanten E., Wisselink G., Friedrich A.W., Kooistra-Smid M., Sinha B., Rossen J.W.A., Couto N. (2019). A comparison of three different bioinformatics analyses of the 16S-23S rRNA encoding region for bacterial identification. Front. Microbiol..

[42] Zhang W., Wei Y., Jin X., Lv X., Liu Z., Ni L. (2022). Spoilage of tilapia by *Pseudomonas putida* with different adhesion abilities. Curr. Res. Food Sci..

[43] Ghosh S.K., Wong M.K.S., Hyodo S., Goto S., Hamasaki K. (2022). Temperature modulation alters the gut and skin microbial profiles of chum salmon (*Oncorhynchus keta*). Front. Mar. Sci..

[44] Ringø E., Sperstad S., Myklebust R., Refstie S., Krogdahl Å. (2006). Characterisation of the microbiota associated with intestine of Atlantic cod (*Gadus morhua L.).* The effect of fish meal, standard soybean meal and a bioprocessed soybean meal. Aquaculture.

[45] Møretrø T., Moen B., Heir E., Hansen A., Langsrud S. (2016). Contamination of salmon fillets and processing plants with spoilage bacteria. Int. J. Food Microbiol..

[46] Kim P.S., Shin N.R., Lee J.B., Kim M.S., Whon T.W., Hyun D.W., Yun J.H., Jung M.J., Kim J.Y., Bae J.W. (2021). Host habitat is the major determinant of the gut microbiome of fish. Microbiome.

[47] Kipp-Sinanis E. (2011). Environmental impact of aquaculture: wild-caught vs. Farmed fish: academic impacts of supplemental instruction and embedded tutors in the classroom view project. https://www.researchgate.net/publication/324165259.

[48] Tavares J., Martins A., Fidalgo L.G., Lima V., Amaral R.A., Pinto C.A., Silva A.M., Saraiva J.A. (2021). Fresh fish degradation and advances in preservation using physical emerging technologies. Foods.

[49] Weihe T., Wagner R., Schnabel U., Andrasch M., Su Y., Stachowiak J., Noll H.J., Ehlbeck J. (2022). Microbial control of raw and cold-smoked atlantic salmon (*Salmo salar*) through a microwave plasma treatment. Foods.

[50] Chen Y., Jackson K.M., Chea F.P., Schaffner D.W. (2001). Quantification and variability analysis of bacterial cross-contamination rates in common food service tasks. J. Food Protect..

[51] Montville R., Chen Y., Schaffner D.W. (2001).

[52] Montville R., Chen Y., Schaffner D.W. (2001). http://www.elsevier.com/locate/ijfoodmicro.

[53] Reij M.W., Den Aantrekker E.D. (2004). Recontamination as a source of pathogens in processed foods. Int. J. Food Microbiol..

[54] Lineback C.B., Nkemngong C.A., Wu S.T., Li X., Teska P.J., Oliver H.F. (2018). Hydrogen peroxide and sodium hypochlorite disinfectants are more effective against *Staphylococcus aureus* and *Pseudomonas aeruginosa* biofilms than quaternary ammonium compounds. Antimicrob. Resist. Infect. Control.

[55] Yi Z., Xie J. (2021). Comparative proteomics reveals the spoilage-related factors of *Shewanella putrefaciens* under refrigerated condition. Front. Microbiol..

[56] Rizzo C., Lo Giudice A. (2022). Life from a snowflake: diversity and adaptation of cold-loving bacteria among ice crystals. Crystals.

[57] Bergholz P.W., Bakermans C., Tiedje J.M. (2009). *Psychrobacter arcticus 273-4* Uses resource efficiency and molecular motion adaptations for subzero temperature growth. J. Bacteriol..

[58] Liao X., Ma Y., Daliri E.B.M., Koseki S., Wei S., Liu D., Ye X., Chen S., Ding T. (2020). Interplay of antibiotic resistance and food-associated stress tolerance in foodborne pathogens. Trends Food Sci. Technol..

[59] Rodríguez-Verdugo A., Lozano-Huntelman N., Cruz-Loya M., Savage V., Yeh P. (2020). Compounding effects of climate warming and antibiotic resistance.

[60] van Dijk H.F.G., Verbrugh H.A., Abee T., Andriessen J.W., van Dijk H.F.G., ter Kuile B.H., Mevius D.J., Montforts M.H.M.M., van Schaik W., Schmitt H., Smidt H., Veening J.-W., Voss A. (2022). Resisting disinfectants. Commun. Med..

[61] Hartmann E.M., Hickey R., Hsu T., Betancourt Román C.M., Chen J., Schwager R., Kline J., Brown G.Z., Halden R.U., Huttenhower C., Green J.L. (2016). Antimicrobial chemicals are associated with elevated antibiotic resistance genes in the indoor dust microbiome. Environ. Sci. Technol..

[62] Li D., Zeng S., He M., Gu A.Z. (2016). Water disinfection byproducts induce antibiotic resistance-role of environmental pollutants in resistance phenomena. Environ. Sci. Technol..

[63] Wood T.K., Knabel S.J., Kwan B.W. (2013). Bacterial persister cell formation and dormancy. Appl. Environ. Microbiol..

[64] Liu Y., Yang K., Zhang H., Jia Y., Wang Z. (2020). Combating antibiotic tolerance through activating bacterial metabolism. Front. Microbiol..

